# Fosmanogepix: The Novel Anti-Fungal Agent’s Comprehensive Review of in Vitro, in Vivo, and Current Insights From Advancing Clinical Trials

**DOI:** 10.7759/cureus.59210

**Published:** 2024-04-28

**Authors:** Ali Almajid, Ali Bazroon, Hashim M Al-awami, Hassan Albarbari, Ibrahim Alqahtani, Rehab Almutairi, Abbas Alsuwayj, Faiza Alahmadi, Jinan Aljawad, Razan Alnimer, Nawal Asiri, Shouq Alajlani, Reem Alshelali, Yamama Aljishi

**Affiliations:** 1 Internal Medicine, King Fahad Specialist Hospital, Dammam, SAU; 2 College of Medicine, Arabian Gulf University, Manama, BHR; 3 College of Medicine, University of Szeged Albert Szent-Györgyi Medical School, Szeged, HUN; 4 College of Medicine, Imam Abdulrahman Bin Faisal University, Dammam, SAU; 5 College of Medicine, Vision College, Riyadh, SAU; 6 College of Medicine, King Khalid University, Abha, SAU; 7 College of Medicine, Umm Al Qura University, Makkah, SAU; 8 Internal Medicine, King Abdullah Medical Complex, Jeddah, SAU

**Keywords:** gwt1., candida, aspergillus, clinical trials, manogepix, e1210, e1211, apx001, fosmanogepix

## Abstract

Fosmanogepix, a prodrug of Manogepix (MGX), is a groundbreaking antifungal agent with broad-spectrum activity against yeasts, including *Cryptococcus* and *Candida*, as well as molds. It exhibits effectiveness against drug-resistant strains, such as *Candida* strains resistant to *echinocandins* and *Aspergillus* strains resistant to azoles. Furthermore, fosmanogepix shows activity against pathogens that typically resist other classes of drugs, such as *Scedosporium*, *Lomentospora prolificans*, and *Fusarium*, although its efficacy against Mucorales varies. In animal models, fosmanogepix has demonstrated notable effectiveness against disseminated infections caused by various *Candida* species, *Coccidioides immitis*, and *Fusarium solani*. It has also shown efficacy in pulmonary infection models involving *Aspergillus fumigatus*, *Aspergillus flavus*, *Scedosporium prolificans*, *Scedosporium apiospermum*, and *Rhizopus arrhizus*. Clinical trials have revealed excellent oral bioavailability (>90%), enabling a seamless transition between intravenous and oral formulations without compromising blood concentrations. Fosmanogepix exhibits favorable profiles in terms of drug interactions, tolerability, and extensive distribution in various tissues, making it an appealing choice for treating invasive fungal infections. This comprehensive review aims to examine the outcomes of published data on fosmanogepix, encompassing in vitro, in vivo, and clinical investigations.

## Introduction and background

Fosmanogepix, a prodrug of Manogepix, is a first-in-class novel antifungal with broad-spectrum in vitro activity against yeasts, namely *Cryptococcus* and *Candida*, as well as molds [1,2]. It works by inhibiting an enzyme called Gwt1, which is involved in the biosynthesis of glycosylphosphatidylinositol (GPI) anchors [3]. The compound butylbenzyl isoquinoline (BIQ) was the first product identified as having the ability to hinder both the growth of fungi and the surface expression of GPI-anchored mannoproteins. The target protein for BIQ was found to be the Gwt1 protein. Subsequently, E1210, the pre-clinical form of manogepix, was discovered to function as a more potent Gwt1 inhibitor. It was licensed in 2015 by Amplyx Pharmaceuticals, and it was later renamed APX001A, then MGX [2,4,5]. The prodrug form, fosmanogepix, is currently under clinical development for the treatment of various invasive yeast and mold infections. Mortality rates from invasive fungal infections vary, with a range of 70% for candidiasis, 50-75% for aspergillosis, and 30-90% for infections caused by other rare molds [6-9]. In light of the morbidity and mortality rates associated with invasive fungal infections, as well as the observed escalation in resistance patterns, an augmented necessity arises for the development of novel antifungal agents characterized by diverse mechanisms of action [10,11]. Fosmanogepix works through a novel mechanism of action, which has been translated into efficacy against various difficult-to-treat and multi-drug-resistant organisms in animal models [12,13]. The current review aims to offer an updated and comprehensive understanding of the novel antifungal agent, fosmanogepix, encompassing its clinical trials and in vitro and in vivo activities.

## Review

Methods

In December 2023, a comprehensive literature search was conducted using multiple databases, including PubMed, Web of Science, and Google Scholar. The search strategy encompassed the following terms: "fosmanogepix," "APX001," "E1211," "E1210," and "manogepix." Initially, 372 citations were identified. The titles of relevant research articles were screened, and full-text articles were retrieved for further evaluation. Studies conducted in vitro, in vivo, or within a clinical context and involving any age group were considered eligible for inclusion in this review.

In vitro activity

Manogepix exhibited extensive efficacy against common *Aspergillus* species, such as *Aspergillus fumigatus* (*A. fumigatus*) and *Aspergillus flavus* (*A. flavus*). The minimum effective concentration (MEC) values consistently demonstrated marked potency, frequently registering below 0.03 mg/L. This signifies a superior level of effectiveness when compared with certain established antifungal agents, such as voriconazole [14]. Moreover, MGX has demonstrated notable efficacy even in the presence of highly resistant molds, including specific strains of *Aspergillus* and *Scedosporium*/*Lomentospora*. Among the antifungal agents under examination, anidulafungin exhibited exceptional efficacy against amphotericin B-resistant strains of *Aspergillus terreus*, boasting a remarkably low minimum effective concentration (MEC90) of just 0.015 μg/ml. Following closely, MGX demonstrated notable effectiveness with an MEC90 of 0.06 μg/ml. In contrast, caspofungin necessitated a higher MEC90 of 0.25 μg/ml. The azoles-posaconazole, itraconazole, and voriconazole manifested significantly lower potency, requiring at least 0.5 μg/ml for 90% inhibition [15]. It is noteworthy that amphotericin B demanded a concentration four times higher (4 μg/ml) than MGX at the MEC90 level. Additionally, MGX displayed striking in vitro efficacy against a diverse range of uncommon and frequently antifungal-resistant fungi, including azole-resistant *Aspergillus* species like *A. lentulus* (MEC 0.008 mg/L) and the *A. ustus* species complex (MEC 0.004-0.015 mg/L) [16]. Nonetheless, MGX's effectiveness against certain strains of *Fusarium* and *Alternaria* was comparatively less pronounced [17]. Furthermore, a study conducted in Spain utilizing the Clinical and Laboratory Standards Institute (CLSI) methods revealed a wider range of MEC values for MGX against *Fusarium* and *Scedosporium*. However, these MEC90 values were comparatively higher than those observed for other species. Significantly, MGX demonstrated superior efficacy when compared to amphotericin B and posaconazole against *L. prolificans*, with MEC values of 0.06 μg/mL, 8 μg/mL, and 16 μg/mL, respectively. Additionally, MGX's effectiveness against the *Fusarium* species complex, with a MEC90 of 0.12 μg/mL, holds promise for addressing these challenging molds known for their resistance to existing therapeutic interventions [17,18].

The comprehensive efficacy of MGX extends across a diverse spectrum of *Aspergillus* species, with a particular highlight being its notable effectiveness against strains resistant to multiple drugs, notably the difficult-to-treat *A. terreus* [15]. While MGX demonstrated robust inhibitory effects against common *Aspergillus* species, its effectiveness diminishes when confronted with *Mucor racemosus* and *Cunninghamella bertholletiae*, necessitating elevated MIC (8 μg/ml and 2-4 μg/ml, respectively). Moreover, MGX's activity experiences further attenuation against dermatophytes such as *Trichophyton rubrum* and *Trichophyton mentagrophytes*, requiring concentrations of 4-8 μg/ml, approximately twice the concentration required for other antifungals [2].

It is noteworthy that approximately 98% of MIC values, as determined through the CLSI and European Committee on Antimicrobial Susceptibility Testing (EUCAST) methods for identical isolates, exhibited concordance. While inconsequential discrepancies were discerned in a limited number of instances, it was observed that the CLSI method generally yielded marginally higher MICs associated with partial inhibition, whereas the EUCAST method manifested slightly elevated MICs indicative of complete inhibition [19]. As part of continuous efforts to discover potent antifungal compounds, the SENTRY antimicrobial surveillance program undertook a research study in 2020 to assess the efficacy of MGX. The investigation encompassed the examination of 1,435 recent clinical fungal isolates sourced globally. This extensive analysis yielded significant insights into the wide-ranging antifungal capabilities of MGX [14]. MGX exhibited remarkable efficacy against the majority of tested *Candida* species, especially *C. albicans* and *C. dubliniensis*. For these particular species, MGX inhibited over 91.7% and 96.6% of isolates at notably low concentrations (0.06 μg/ml and 0.25 μg/ml, respectively). However, its effectiveness was comparatively reduced against *C. kefyr* and *C. krusei*, the latter demonstrating intrinsic resistance. Nevertheless, MGX continued to inhibit a substantial proportion of these *Candida* species [20]. A study conducted by Maphanga et al. investigated the efficacy of MGX against *Candida auris* isolates, particularly those resistant to other antifungal agents. Out of 394 *C. auris* isolates, 91% showed resistance to at least one antifungal class. Most notably, 335 isolates were resistant to fluconazole alone. MGX demonstrated low MIC values against these isolates, ranging from 0.002mg/mL to 0.063mg/mL. Additionally, 19 isolates resistant to both fluconazole and amphotericin B exhibited low MGX MICs. Two isolates from the same patient were resistant to all three antifungal classes, and their MGX MICs were also low. Using whole-genome sequencing (WGS), *C. auris* was classified into four genetic clades based on their geographic origin: clade I (South Asia), clade II (East Asia), clade III (Africa), and clade IV (South America) [21]. Specific amino acid substitutions were identified in some isolates, such as VF125AL and Y132F in Erg11p for clade III and clade I isolates, respectively. Echinocandin-resistant clade III isolates had the S639P substitution in Fks1p [22]. When examining *C. auris* clades, 70 out of 84 sequenced isolates belonged to clade III, and all were resistant to at least one antifungal agent. Despite clade differences, all 84 sequenced isolates showed low MGX MICs [22]. Overall, MGX demonstrated activity against a wide range of *C. auris* isolates, including those resistant to other antifungal drugs [22]. When comparing the antifungal effectiveness of MGX to other options such as fluconazole, itraconazole, amphotericin B, and micafungin, MGX stands out prominently due to its superior antifungal potency. It achieves levels of effectiveness comparable to voriconazole [2]. In the case of six isolates of *C. auris* that demonstrated resistance to multiple drugs, MGX consistently displayed strong effectiveness. The MIC values for these isolates varied from 0.008 mg/liter to 0.015 mg/liter, with a median and mode of 0.015 mg/liter [23].

When tested against *C. neoformans var. grubii*, MGX exhibited a broader spectrum of activity in comparison to *Candida spp.*, with MICs ranging across nine 2-fold dilution steps (0.015-4 mg/L). Nevertheless, the inhibitory effect persisted in 95.9% of isolates at concentrations of 2 mg/L or lower [16]. *Cryptococcus neoformans* is a significant cause of life-threatening meningitis. Research has demonstrated fosmanogepix’s high efficacy as a standalone treatment and even greater potency when employed in conjunction with another antifungal medication, namely fluconazole [24]. In laboratory models, this combination resulted in a notable reduction in fungal burden, providing optimism for the development of more effective treatment alternatives [24].

Mycetoma, primarily caused by *Madurella Mycetomatis* (*M. mycetomatis*), is a tropical disease frequently overlooked. Nonetheless, it remains a major concern in tropical countries [25,26]. The management of mycetomas of fungal origin continues to pose challenges, primarily attributable to the restricted therapeutic alternatives [27,28]. The study conducted by Konings et al. presents a comprehensive investigation into the potential of MGX in inhibiting the growth of *M. mycetomatis*, exploring its synergistic effects with itraconazole, and evaluating its in vivo efficacy using a Galleria mellonella mycetoma model [29]. The researchers utilized MGX and itraconazole, along with 22 clinical isolates of *M. mycetomatis* sourced from various regions. The study employed a range of techniques, including in vitro susceptibility testing and analysis of synergism between manogepix and itraconazole. The Galleria mellonella grain model was used for assessing in vivo efficacy. The results demonstrated that MGX exhibited inhibitory effects on *M. mycetomatis* growth in vitro, with MICs ranging from <0.008 to 16 mg/l. Despite the diverse MICs, genetic analysis did not reveal a clear link between high and low MICs. Synergism between MGX and itraconazole was observed for the majority of isolates. However, in the Galleria mellonella model, both monotherapy and combination therapy failed to significantly improve larval survival [29].

Mechanism of action

Fosmanogepix is an N-phosphonooxymethyl prodrug that undergoes metabolic conversion by systemic phosphatase, resulting in the formation of the active moiety MGX [3,30,31]. MGX functions through the inhibition of the Gwt1 protein. Gwt1 is a conserved fungal enzyme pivotal for catalyzing inositol acylation, a critical initial step in the glycosylphosphatidylinositol-anchor biosynthesis pathway [3]. Gwt1 plays a vital role in the trafficking and anchoring of mannoproteins to the outer cell wall and cell membrane [5]. Mannoproteins play a crucial role in maintaining cell wall integrity, adhesion, pathogenicity, and evasion of the immune system. Studies have demonstrated that the maturation and localization of GPI-anchored mannoproteins are influenced by the inhibition of Gwt1 in both *Candida albicans* and *Saccharomyces cerevisiae* [3]. The specificity of MGX activity seems to be confined to fungal pathogens [3].

Pre-clinical pharmacokinetic

Fosmanogepix can be administered via oral or intravenous routes, demonstrating substantial oral bioavailability exceeding 90%. In animal models, significant concentrations of the compound are achieved in both ocular and central nervous system tissues [31]. Rats and monkeys receiving fosmanogepix via oral or intravenous administration displayed prompt and extensive absorption across diverse tissues, encompassing the lung, brain, liver, kidney, and eye. The principal elimination routes were identified as biliary in rats and fecal in monkeys [32]. Assessing efficacy through the utilization of a rigorously validated model of disseminated candidiasis, the findings of the study by Arendrup et al. revealed a robust correlation between efficacy and critical pharmacokinetic/pharmacodynamic (PK/PD) parameters. Specifically, the free fraction area under the plasma concentration-time curve over minimum inhibitory concentration (fAUC/MIC) and the free fraction maximum (peak) plasma drug concentration over MIC (fCmax/MIC) showed corresponding R2 values of 0.88 and 0.83, respectively. In PK/PD investigations employing a disseminated infection model for *Candida albicans*, *Candida glabrata*, and *Candida auris*, the median free drug AUC/MIC PK/PD targets for stasis were determined as 191.59, 11.48, and 99.69, respectively. Notably, these targets were identified across multiple strains of each *Candida* species, emphasizing the significance of considering strain variability in PK/PD assessments [33].

In vivo effectiveness

In a comprehensive investigation by Petraitiene et al., the effectiveness of fosmanogepix in a rabbit model of *Candida endophthalmitis* and hematogenous *Candida meningoencephalitis* (HCME) was thoroughly examined [31]. The study involved assessing the decrease in fungal burden through colony-forming unit (CFU) count analysis and monitoring (1→3)-β-d-glucan levels as a biomarker for the therapeutic response. Significant reductions (1 to >2 log10) in the residual fungal burden of *C. albicans* were evident in the choroid and vitreous humor of rabbits treated with fosmanogepix or amphotericin B deoxycholate (DAMB), highlighting comparable efficacy between the two treatments. The research emphasized a consistent reduction (>2.0 log10) in residual fungal burden across various central nervous system (CNS) tissues and cerebrospinal fluid (CSF) at all fosmanogepix dosages, as well as in DAMB-treated rabbits, highlighting the broad effectiveness of fosmanogepix in diminishing fungal burden. The evaluation of (1→3)-β-d-glucan levels as a biomarker for therapeutic response revealed substantial reductions in CSF of rabbits treated with fosmanogepix at 50 mg/kg twice daily, and 100 mg/kg twice daily, or DAMB at 1 mg/kg once daily (QD). A concurrent reduction in (1→3)-β-d-glucan levels was observed in serum for rabbits treated with fosmanogepix and DAMB, with a more pronounced reduction in serum than in CSF, indicating compartmentalization within the CSF. These findings underscored the promising efficacy of fosmanogepix in diminishing both fungal burden and (1→3)-β-d-glucan levels in a rabbit model of *Candida endophthalmitis* and HCME, highlighting its potential as a valuable antifungal agent. In the investigation, untreated infected control rabbits demonstrated notable increases in serum creatinine levels in comparison to those subjected to fosmanogepix at doses of 25 mg/kg and 50 mg/kg (P < 0.01). No notable distinctions were discerned in serum creatinine levels among the various fosmanogepix dosage groups. Rabbits treated with fosmanogepix at 25 mg/kg, 50 mg/kg, and 100 mg/kg exhibited significant reductions in serum urea nitrogen when contrasted with untreated rabbits (P ≤ 0.05). In contrast, animals treated with DAMB displayed marked increases in both serum creatinine and serum urea nitrogen, indicative of its acknowledged nephrotoxicity. Serum potassium levels remained constant across all treatment groups and untreated control rabbits. Neither fosmanogepix-treated rabbits nor DAMB-treated rabbits demonstrated an increase in serum hepatic transaminase levels, while untreated infected control rabbits manifested a noteworthy elevation in alanine aminotransaminase (ALT) and aspartyl aminotransaminase (AST) compared to all treatment groups (P < 0.01) [31].

Alkhazraji et al. explored the effectiveness of MGX in treating lethal invasive fungal infections induced by *Fusarium* and *Scedosporium* in immunosuppressed mice [34]. They evaluated the in vitro activity of MGX, discovering an MEC of 0.03 μg/ml for *Scedosporium* and a range of 0.015 to 0.03 μg/ml for *Fusarium* isolates. MGX treatment, in conjunction with 1-aminobenzotriazole (ABT), to extend MGX's serum half-life significantly prolonged median survival times compared to a placebo [34]. Additionally, the investigation illustrated that MGX’s efficacy was comparable to a clinically relevant high dose of liposomal amphotericin B survival rates markedly improved with MGX treatment, and the decrease in tissue fungal burden remained consistent across different doses, indicating a dose-response correlation. The researchers utilized quantitative (qPCR) to quantify log10 conidial equivalents/gram of tissue, unveiling a dose-dependent reduction in fungal burden in response to MGX treatment. Furthermore, mice treated with MGX exhibited survival rates and tissue clearance comparable to those administered liposomal amphotericin B [34].

In a study investigating the effectiveness of fosmanogepix in treating pulmonary mucormycosis caused by *Rhizopus arrhizus*, the researchers began by evaluating the antifungal susceptibility of MGX, posaconazole (POSA), and isavuconazole (ISA) against clinical isolates of two *Rhizopus arrhizus varieties-var* [35]. *Arrhizus* and *var. delemar*'s varying susceptibility patterns were noted, with *R. arrhizus var. delemar* exhibiting greater sensitivity to MGX than *R. arrhizus var. arrhizus*. In the subsequent phase, the researchers employed a highly immunosuppressed murine model to assess the efficacy of fosmanogepix in treating pulmonary mucormycosis caused by *R. arrhizus var. delemar*. Immunocompromised mice were treated with different doses of fosmanogepix, ISA, or a placebo. The results demonstrated that fosmanogepix, especially at clinically relevant exposures (78 mg/kg and 104 mg/kg), significantly improved the survival rates of the mice compared to the placebo control. The study also noted a dose-dependent prolongation in the median survival time of mice treated with fosmanogepix. Furthermore, the researchers evaluated the tissue fungal burden in the lungs and brains of mice using qPCR at day +4 post-infection. Fosmanogepix treatment resulted in a substantial reduction in fungal burden, as evidenced by a notable decrease in conidial equivalents/gram of tissue. The highest dose of fosmanogepix (104 mg/kg) exhibited superior efficacy in reducing tissue fungal burden compared to the lower dose (78 mg/kg) and was comparable to ISA treatment. The study emphasized the potential of fosmanogepix as an effective treatment for pulmonary mucormycosis caused by *R. arrhizus var. delemar* [35].

Wiederhold et al. investigated the efficacy of delayed therapy with fosmanogepix in a murine model of *Candida auris* invasive *candidiasis*, focusing on fosmanogepix's antifungal effectiveness and its impact on survival [36]. Neutropenic mice were intravenously infected with a fluconazole-resistant clinical isolate of *Candida auris*. Treatment commenced 24 hours post-inoculation, involving various regimens: control (no therapeutic agent), fosmanogepix (104 and 130 mg/kg intraperitoneally three times daily or 260 mg/kg intraperitoneally twice daily), fluconazole (20 mg/kg orally once daily), or caspofungin (10 mg/kg intraperitoneally once daily), administered continuously for seven days. The fungal burden was evaluated by counting colonies in the kidneys and brains on day eight. In the survival analysis, mice treated with fosmanogepix at clinically relevant doses (104 mg/kg and 130 mg/kg intraperitoneal three times daily or 260 mg/kg intraperitoneal twice daily) showed notably higher median survival and percent survival in fosmanogepix-treated groups compared to the control (P < 0.0001) [36-38]. Fungal burden assessment revealed a significant reduction in kidney fungal burden in mice treated with high-dose fosmanogepix (260 mg/kg twice daily) and caspofungin (10 mg/kg/day). Brain tissue fungal burden was also notably reduced in these groups, indicating the effectiveness of both agents in controlling the infection. Importantly, these reductions exceeded 1 log10 CFU/g compared to pre-therapy levels, further supporting the efficacy of fosmanogepix in controlling *Candida auris* infection in this murine model [36].

Case studies 

*Aspergillus calidoustus* is an azole-resistant, cryptic species within the *Aspergillus* genus, known for its association with an elevated mortality rate. It is frequently observed in immunosuppressed patients and commonly presents as an extrapulmonary infection [39]. Camargo et al. published a case report detailing a patient infected with multi-drug-resistant *Aspergillus calidoustus*, exhibiting disseminated infection. The case involved a 40-year-old male with a history of hematopoietic cell transplantation for relapsed Hodgkin's lymphoma, complicated by chronic graft-versus-host disease (GvHD) requiring immunosuppression. Despite prophylaxis with isavuconazole, the patient exhibited elevated serum galactomannan and chest CT findings suggestive of a fungal etiology. Despite switching to voriconazole, the patient's condition worsened, leading to bronchoscopy and the revelation of *Aspergillus galactomannan*. Imaging also revealed brain lesions, indicating possible fungal cerebritis. Subsequent antifungal treatments, including micafungin, liposomal amphotericin B, and voriconazole, were not fully effective. The patient's condition continued to deteriorate, prompting the addition of fosmanogepix under emergency investigational new drug (EIND) authorization. Fosmanogepix was administered alongside other antifungals, and the patient underwent mitral valve vegetectomy due to infective endocarditis. Postoperatively, the patient was switched to oral fosmanogepix, leading to a decline in serum galactomannan levels. Despite an episode of femoral artery thrombosis and surgical confirmation of an *Aspergillus* infection, the patient showed improvement. Liposomal amphotericin B was discontinued, and serum galactomannan eventually became negative. The patient transitioned to fosmanogepix monotherapy, tolerating it well with no adverse effects and maintained negative serum galactomannan levels at the last clinic follow-up on day 324. Antifungal susceptibility testing for *Aspergillus calidoustus* indicated resistance to various agents, except fosmanogepix, which displayed a low MIC (≤0.008 μg/mL). Synergy testing also demonstrated favorable interactions between fosmanogepix and other antifungal agents. The patient's response to fosmanogepix, especially in the context of multi-drug resistance, suggests its potential as a therapeutic option for challenging invasive fungal conditions [40].

Infections caused by *Fusarium* species in immunocompromised individuals often lead to widespread dissemination with a high mortality rate, approaching as high as 90%, despite the utilization of currently available antifungals [41-44]. Winston et al. presented a case detailing central nervous system fusariosis in a patient with prolonged neutropenia [45]. A 26-year-old undergoing chemotherapy for relapsed acute myelogenous leukemia (AML) faced persistent fevers, exacerbated brain parenchymal lesions on magnetic resonance imaging (MRI), and a positive blood culture revealing *Fusarium lactis* resistance to azoles and echinocandins but sensitivity to MGX. Initial treatment with caspofungin, followed by liposomal amphotericin B plus voriconazole, yielded no improvement. On day 20, the therapeutic strategy shifted to fosmanogepix combined with liposomal amphotericin B, resulting in notable clinical improvement. Subsequent brain MRIs demonstrated reductions in both the size and intensity of parenchymal lesions. The absolute neutrophil count gradually rose, and a bone marrow biopsy confirmed AML remission. Oral fosmanogepix was continued for six months, followed by allogeneic hematopoietic stem cell transplantation. Ten months post-transplantation, fosmanogepix was successfully discontinued, with no evidence of recurrent fusariosis [45].

Goggin et al. reported a case detailing the successful treatment of cutaneous fusariosis resistant to conventional therapies [46]. In this notable case study, a 14-year-old male with STAT3 autosomal dominant hyper-immunoglobulin E syndrome (STAT3 AD-HIES) and end-stage kidney disease (ESKD) was diagnosed with persistent cutaneous *Fusariosis* at the age of three. Despite eight years of intermittent and unsuccessful antifungal treatments, including IV liposomal amphotericin B and various combinations, the *Fusarium solani* species complex (FSSC) infection on his right forearm persisted and extended from the wrist to the elbow. The patient, who had experienced acute kidney injury due to amphotericin, had lost follow-up and was hypertensive at age 12. Further treatment was initiated using fosmanogepix obtained through an expanded access program. The patient faced challenges with medication tolerance and adherence, experiencing nausea and vomiting initially. Adjustments were made to the fosmanogepix dosage, and the patient's symptoms resolved. After approximately six months of fosmanogepix therapy, skin biopsies turned negative for FSSC, marking a successful outcome. This case underscores the efficacy of fosmanogepix in treating a challenging cutaneous *Fusariosis* in a pediatric patient with complex medical conditions, shedding light on the successful management of a rare and highly resistant fungal infection in the context of immunodeficiency and ESKD [46]. Table 1 summarizes the aforementioned cases.

**Table 1:** Summary of case reports on Fosmanogepix. HSCT: Hematopoietic stem cell transplantation; HL: Hodgkin's lymphoma; GVHD: Graft-versus host disease; AML: Acute myeloid leukemia; FMGX: Fosmanogepix; AMB: Amphotericin B

Author (s)	Case summary	Duration of Therapy	Outcome
Camargo et al. [40]	A 40-year-old male who underwent HSCT for Hodgkin's lymphoma (HL) experienced complications with graft-versus-host disease (GvHD) and subsequently developed a disseminated infection with multi-drug-resistant Aspergillus calidoustus	It is approximately 191 days, though it is not explicitly mentioned	Significant improvement with negative serum galactomannan
Winston et al. [45]	A 26-year-old with AML faced a Fusarium lactis infection, resistant to azoles and echinocandins. FMGX combined with liposomal AMB	6 months	Clinical improvement, reductions in brain lesions, and an absolute neutrophil count rise, resulting in AML remission
Goggin et al. [46]	A 14-year-old with immunodeficiency and end-stage kidney disease has had persistent cutaneous Fusariosis since age 3. Despite prior unsuccessful treatments, FMGX was successful, leading to negative skin biopsies	6 months	Clinical improvement with negative skin biopsies

Clinical trials

In the phase 1 study evaluating the safety and pharmacokinetics of single and multiple oral doses of fosmanogepix and investigating the impact of food on its bioavailability, participants underwent a comprehensive regimen [37]. This included receiving single intravenous doses of 200 mg over three hours, followed by single tablet doses ranging from 100 to 500 mg, with a 14-day washout period between each dose escalation. Cohort 1b, comprising 10 subjects randomized in an 8:2 ratio for fosmanogepix or placebo, received a single oral tablet dose of 400 mg under both fed and fasted conditions, with a 14-day washout period. Multiple ascending dose (MAD) cohorts, each with eight subjects randomized in a 6:2 ratio for fosmanogepix or placebo, received oral tablet doses of 500 and 1000 mg daily for 14 days. The pharmacokinetic analysis demonstrated that plasma exposure to APX001A displayed linearity, dose proportionality, low intersubject variability, and a half-life of approximately 2.5 days. Accumulation of APX001A was observed in the MAD cohorts, with AUC0-24 values of 192 and 325 µg·hours/mL after 14 days of dosing at 500 and 1000 mg, respectively. The oral bioavailability surpassed 90%. Importantly, administering fosmanogepix with a high-fat, high-calorie meal did not impact the rate or extent of absorption [37].

Hodges et al. published the first study of fosmanogepix in humans, aimed at evaluating its safety and pharmacokinetics [38]. Administered through intravenous infusion to a group of healthy participants, the trial included six cohorts with single ascending doses (SAD) and four cohorts with multiple ascending doses (MAD), each comprising eight subjects. A randomized allocation in a 6:2 ratio ensured participants received either three-hour intravenous infusions of APX001 or a placebo. SAD cohorts (1-6) underwent the administration of single doses ranging from 10 to 350 mg, while MAD cohorts (7-10) received daily doses of 50, 150, 300, and 600 mg, respectively, over 14 days. Pharmacokinetic parameters for APX001A in plasma were meticulously elucidated using non-compartmental analysis. The study outcomes revealed noteworthy linearity, dose proportionality, and low intersubject variability in APX001A plasma exposure, coupled with an approximately 2.5-day half-life. In alignment with the expected effects based on dosing frequency and half-life, MAD cohorts demonstrated a discernible accumulation of APX001A. Notably, a single 350 mg dose of APX001 sustained drug levels surpassing the minimum inhibitory concentration (MIC) for *Candida* and *Aspergillus* for a week. On the 14th day, the area under the curve (AUC0-24) for the 600 mg/day dose reached 245 µg·hours/mL. APX001 exhibited commendable tolerability across all administered doses, with no clinically significant adverse events except for one subject discontinuing due to flu. Remarkably, no dose-limiting toxicities were observed, and the majority of adverse events proved mild and transient and necessitated no intervention, with headache emerging as the most prevalent occurrence [38].

In the Phase 1b study published by Cornely et al., which focused on evaluating the safety, tolerability, and pharmacokinetics of fosmanogepix in neutropenic patients with acute myeloid leukemia (AML) undergoing remission induction chemotherapy [47], the trial included intravenous (IV) and oral administration of fosmanogepix in two cohorts. Safety assessment, the primary endpoint, involved monitoring adverse events (AEs), physical examinations, and laboratory tests. The study included 21 participants (10 IV, 11 oral) for safety analysis and 18 for pharmacokinetic analysis. Results indicated that both IV and oral fosmanogepix were well-tolerated. A total of 26 adverse events (AEs) related to fosmanogepix were observed in nine participants (42.9%). These events were generally mild to moderate, with none being serious or leading to the discontinuation of the study drug. The most common fosmanogepix-related AE was nausea, occurring in three patients (14.3%). Other AEs included vomiting, an increase in ALT, and delirium, each observed in two patients (9.5%). Vomiting and delirium were found in the cohort receiving intravenous (IV) fosmanogepix. Two instances of ALT increase were reported, one preceding febrile neutropenia and sepsis and the other following *Klebsiella pneumoniae* infection and pyrexia. Both ALT increase events were resolved, and the majority of fosmanogepix-related events were recovering or recovered by the last assessment. One participant in the IV cohort had fosmanogepix administration interrupted due to three chills events, possibly related to the drug [47].

A phase 2 clinical trial was conducted to evaluate the clinical safety and effectiveness of fosmanogepix in the treatment of candidaemia [48]. Eligible participants, identified with candidaemia through a positive blood culture for *Candida spp.,* within 96 hours before study entry and having received no more than two days of systemic antifungals, were administered fosmanogepix for 14 days. The treatment started with 1000 mg intravenously twice daily on day 1, followed by a maintenance dose of 600 mg intravenously once daily, allowing an optional switch to 700 mg orally once daily from day four onwards. The modified intent-to-treat (mITT) population, comprising eligible participants who received at least one dose of fosmanogepix and had a confirmed *candidaemia* diagnosis within 96 hours of initiating treatment, demonstrated an 80% treatment success rate (16 out of 20) according to the end-of-study (EOST) assessment. The survival rate on day 30 was 85% (17 out of 20), with three deaths unrelated to fosmanogepix. Approximately 48% (10 out of 21) of participants transitioned to oral fosmanogepix, and notably, the treatment exhibited good tolerance with no treatment-related serious adverse events or discontinuations [48].

Another phase 2 trial was conducted to evaluate the clinical efficacy and safety of fosmanogepix in patients with candidemia caused by *Candida auris* [49]. Eligible participants, aged 18 and above, diagnosed with candidemia or invasive candidiasis from *C. auris*, showing clinical signs and having limited treatment options, were administered fosmanogepix, with the option to switch to oral fosmanogepix 800 mg once daily from day 4. The primary endpoint, evaluated by an independent data review committee (DRC), was treatment success at the end of the study treatment (EOST). In a cohort of nine participants with candidemia in South African intensive care units, all received only intravenous fosmanogepix. The DRC-assessed treatment success at EOST and the day 30 survival rate were 89% (eight out of nine). No treatment-related adverse events or study drug discontinuations were reported. These findings suggest that fosmanogepix is safe, well-tolerated, and effective for candidemia caused by *C. auris* [49].

Upcoming clinical trials

A clinical trial was conducted to evaluate the efficacy of fosmanogepix in treating invasive mold infections, such as *Aspergillus* or rare molds (NCT04240886). The primary objectives of the trial were to assess the safety of the drug and its potential to decrease mortality rates. The study was conducted as an "open-label" trial, with no randomization. It involved 21 patients from four different countries, with 20 cases having either probable invasive mold infection (IMI) or proven IMI from different sites in the body (lung, sinuses, and other parts of the body). Participants received the drug either orally or intravenously over six weeks. Subsequent evaluations took place four weeks after the last dose of fosmanogepix. The study's findings suggested that fosmanogepix could be a viable option for treating invasive mold infections, with a notable decrease in the mortality rate from 45% to 25% compared to other antifungal medications used in different trials. The trial was terminated earlier than the agreed-upon timeframe because the sponsor intended to conduct a larger study on the drug. The termination did not raise any safety concerns (NCT04240886).

Other completed trials aimed to study the drug's bioequivalence (NCT05491733) and its interaction with itraconazole and rifampin (NCT04166669), although results have not been published yet. Anticipated future trials include Phase 3 treatment of candidemia and/or invasive candidiasis (NCT05421858) and the use of fosmanogepix in patients with hepatic dysfunction (NCT05582187).

## Conclusions

In conclusion, both MGX and its prodrug, fosmanogepix, exhibit significant promise as antifungal agents, addressing a spectrum of challenging fungal infections. MGX demonstrated notable efficacy against various *Aspergillus* and *Candida* species, highlighting its potential therapeutic value, especially in the context of drug-resistant strains. The drug's specific mechanism of action, targeting the Gwt1 protein critical for glycosylphosphatidylinositol-anchor biosynthesis, underscores its fungal pathogen specificity. Pre-clinical pharmacokinetic studies for MGX reveal favorable oral bioavailability and extensive tissue distribution, including the central nervous system and ocular tissues. In vivo effectiveness studies across diverse infection models consistently highlight MGX's potent antifungal activity, reinforcing its potential clinical significance. Similarly, fosmanogepix showed clinically promising results in managing invasive fungal infections caused by *Aspergillus calidoustus* and *Fusarium* species, particularly in cases resistant to conventional therapies. Its effectiveness in challenging infections, such as central nervous system fusariosis and cutaneous fusariosis, positions fosmanogepix as a valuable addition to the antifungal armamentarium. Phase 1 trials demonstrated its safety, tolerability, and pharmacokinetics. The phase 2 trials investigating fosmanogepix in candidemia, including cases caused by *Candida auris*, demonstrated high treatment success rates and good tolerance, indicating potential broad applicability across various fungal infections. Collectively, the evidence supports fosmanogepix as a promising antifungal agent, offering hope for improved management of various fungal infections, especially those caused by drug-resistant strains. Further clinical studies are essential to validate these findings and establish fosmanogepix as a valuable agent in combating fungal infections.
